# Effect of cognitive reserve on the association between slow wave sleep and cognition in community-dwelling older adults

**DOI:** 10.18632/aging.204943

**Published:** 2023-09-28

**Authors:** Valentin Ourry, Stéphane Rehel, Claire André, Alison Mary, Léo Paly, Marion Delarue, Florence Requier, Anne Hendy, Fabienne Collette, Natalie L. Marchant, Francesca Felisatti, Cassandre Palix, Denis Vivien, Vincent de la Sayette, Gaël Chételat, Julie Gonneaud, Géraldine Rauchs, the Medit-Ageing Research Group

**Affiliations:** 1 Normandie University, UNICAEN, INSERM, U1237, Physiopathology and Imaging of Neurological Disorders (PhIND), Institut Blood and Brain @ Caen-Normandie, Cyceron, France; 2 Normandie University, UNICAEN, PSL Université, EPHE, INSERM, U1077, CHU de Caen, GIP Cyceron, NIMH, Caen, France; 3 Neuropsychology and Functional Imaging Research Group (UR2NF), Centre for Research in Cognition and Neurosciences (CRCN), UNI - ULB Neuroscience Institute, Bruxelles 1050, Belgium; 4 University of Liege, GIGA CRC Vivo Imaging, Liege, Belgium; 5 University of Liege, Psychology and Neuroscience of Cognition, Liege, Belgium; 6 Division of Psychiatry, University College London, London, United Kingdom; 7 Département de Recherche Clinique, CHU de Caen, Caen, France; 8 Service de Neurologie, CHU de Caen, Caen, France

**Keywords:** sleep, aging, cognitive reserve, cognition

## Abstract

Sleep, especially slow wave sleep (SWS), is essential for cognitive functioning and is reduced in aging. The impact of sleep quality on cognition is variable, especially in aging. Cognitive reserve (CR) may be an important modulator of these effects. We aimed at investigating this question to better identify individuals in whom sleep disturbances might have greater behavioral consequences. Polysomnography and neuropsychological assessments were performed in 135 cognitively intact older adults (mean age ± SD: 69.4 ± 3.8y) from the Age-Well randomized controlled trial (baseline data). Two measures of cognitive engagement throughout life were used as CR proxies. Linear regression analyses were performed between the proportion of SWS, and executive function and episodic memory composite scores. Then, interaction analyses between SWS and CR proxies on cognition were conducted to assess the possible impact of CR on these links. SWS was positively associated with episodic memory, but not with executive function. CR proxies modulated the associations between SWS and both executive and episodic memory performance. Specifically, individuals with higher CR were able to maintain cognitive performance despite low amounts of SWS. This study provides the first evidence that CR may protect against the deleterious effects of age-related sleep changes on cognition.

## INTRODUCTION

Sleep disturbances are a frequent complaint in older adults, affecting more than a half of older individuals [1]. This complaint is confirmed by objective changes as evidenced in polysomnography studies, which reveal longer sleep onset latency, shorter total sleep duration, increased sleep fragmentation, and a reduced amount of deeper non-rapid eye movement (NREM) sleep, also known as slow wave sleep (SWS, or N3 stage), contrasting with an increase in lighter NREM sleep stages N1 and N2 in older adults [2]. Changes in REM sleep also occur with aging, but they are subtler than those of NREM sleep and appear later in life [2].

A number of studies have investigated the impact of sleep changes on cognitive performance and dementia risk, providing inconsistent findings. Different methodological approaches have been used, ranging from large epidemiological studies using self-reports of sleep quality, to more specific experimental designs using polysomnography. Various aspects of sleep including poor self-reported sleep quality, short sleep duration and sleep fragmentation have been quite consistently associated with an increased risk of cognitive decline and AD dementia (see [3, 4] for systematic reviews and meta-analyses; [5, 6]). However, cross-sectional studies assessing the association between sleep quality and cognition in older adults yielded inconsistent results. For instance, concerning sleep duration, both self-reported short and long sleep durations have been previously associated with reduced cognitive function in older adults [7], but not systematically ([8] for a review and meta-analysis), and the same is true when using an objective measure of total sleep time obtained from actigraphy (e.g., [9]). The links between sleep duration and cognition may also depend on the cognitive domain assessed, with significant associations reported for executive function, verbal memory and working memory, but not for processing speed [8]. Similarly, for sleep fragmentation, increased time spent awake during the night was associated with poorer delayed recall performance [10], but the opposite pattern of results has also been observed [11].

SWS is markedly affected by the aging process. SWS-related parameters, including reduced Slow Wave Activity (SWA; corresponding to the spectral power of slow waves), have been associated with poorer executive functioning [12, 13] and episodic memory [14, 15] in older adults. Interestingly, a recent randomized control trial showed that auditory tones used to enhance SWS improved executive function in middle-aged men confirming, with another methodological approach, the potential role of SWS on cognitive performance [16]. However, several studies failed to report a significant association between SWS and cognition. For instance, Groeger et al. reported that SWS disruption, using acoustic stimulation, had small effects on executive function in young, middle-aged and older adults, with no between group differences [17]. In another study analyzing data collected in more than 200 young, middle-aged and older adults, no significant associations were found between SWS and executive function, challenging the view that age-related changes in SWS are a major driver of executive function decline in aging [18]. Overall, it appears that the association between sleep and cognition is weaker in older adults compared to younger individuals [19].

Above methodological differences in terms of age range, sample sizes and the type of sleep measures used (subjective vs objective), the heterogeneity of previous results could be explained by different capacities of older individuals to tolerate the effects of sleep disruption. Indeed, older adults appear to better tolerate the effects of sleep deprivation than their younger counterparts [20], suggesting the development of compensatory mechanisms throughout life [19]. However, the factors promoting these capacities remain to be unraveled. Cognitive reserve (CR) is a concept developed to account for the heterogeneous associations between brain alterations and cognitive deficits, with some individuals being able to maintain good cognitive performance despite the presence of significant brain alterations [21]. Possibly, differences in CR could also account for the heterogeneous associations between sleep disturbances and cognitive deficits. CR is determined by genetic and environmental factors and nurtured by exposure to mental activities throughout life [21], and it may slow down cognitive decline [22] and reduce dementia risk [23]. CR is a complex construct that is often indirectly estimated using various proxies such as education attainment, intelligence quotient (IQ), occupation complexity or leisure activities [24]. Although education is not a perfect measure (i.e., related to a specific time frame and unvarying) [25], it is the most widely used as CR proxy [26]. However, as CR is a dynamic process and is influenced by genetic and environmental factors across the lifespan, there is a need to better assess lifestyle throughout life. To provide more detailed measures that take into account the life-course and the richness of experiences that nurture CR, several psychometric instruments have been developed, such as the Cognitive Activities Questionnaire [27] and the Lifetime of Experiences Questionnaire [28].

The potential impact of CR in the context of sleep disturbances has hardly been explored. In a study conducted in patients with obstructive sleep apnea, in which CR was assessed using IQ, Alchanatis et al. [29] reported that, while patients with obstructive sleep apnea and low CR had lower attention/alertness performance than IQ-matched controls (without obstructive sleep apnea), patients with higher CR had similar performance compared to their IQ-matched controls. This suggests that CR may have a protective effect against sleep apnea-related cognitive deficits. In the same vein, a few other cross-sectional studies have shown an effect of education on the link between self-reported sleep difficulties or nocturnal awakenings and executive function [30, 31]. These studies showed that CR might moderate the impact of sleep disturbances on cognition. However, there are inconsistent results as well, which may also be due to methodological differences. For instance, in the study by Yeh et al. [32] assessing the association between sleep latency or wake after sleep onset and episodic memory, the CR proxy used was a reading/pronunciation score for increasingly unfamiliar and phonologically difficult words, which was less common than the measures used in the other studies and potentially influenced by various cognitive processes making this test a more biased proxy of CR. Finally, to the best of our knowledge, no study to date has evaluated the potential modulation of the relationship between sleep disturbances and cognition using both polysomnography, the gold-standard for objective measurement of sleep, and questionnaires capturing the complexity and richness of CR throughout life.

Given that SWS is markedly reduced in aging [2] and has been associated with executive function [12, 13] and memory processes [14, 15], including sleep-dependent memory consolidation [33], we investigated whether CR proxies assessed along the life-course modulate the impact of sleep changes in older-age, especially the decrease in SWS, on cognition. In a group of 135 cognitively unimpaired older adults from the Age-Well cohort [34] who underwent a polysomnography, a neuropsychological battery and a lifestyle assessment, we first examined the associations between the proportion of SWS and both executive function and episodic memory, which are known to be particularly sensitive to aging and Alzheimer’s disease [35]. Then, we investigated whether CR proxies (engagement in complex mental activities throughout the life course) moderates the association between SWS and cognition. In addition, exploratory analyses were conducted to determine whether there is a life period during which building CR might be more important to overcome the effect of age-related sleep changes. We hypothesized that CR may reduce the effect of sleep changes in older-age on cognition and that the exposure to enriched lifestyle could be more influential at certain life periods.

## RESULTS

### Associations between demographics, sleep, cognition and CR proxies

In 135 cognitively unimpaired older adults from the Age-Well cohort aged 65 and above (mean age = 69.4 ± 3.8; 83 women), a polysomnography was acquired and allowed to obtain the proportion of SWS relative to total sleep time. A neuropsychological battery allowed to compute an episodic memory and an executive function composite score (see Methods for details). Finally, the number of years of schooling (education), the Cognitive Activities Questionnaire (CAQ) [27] and the Lifetime of Experiences Questionnaire (LEQ) [28, 36] were used to estimate CR. Both the CAQ and LEQ assessed cognitive and complex mental activities across the life-course, providing proxies of early-life, mid-life, late-late and lifelong (total) CR. Demographics and observed scores are detailed in Table 1. Age was negatively associated with the executive function composite score (r = −0.26, *p* = 0.002), the episodic memory composite score (r = −0.19, *p* = 0.024) and education (r = −0.18, *p* = 0.042). Age was not associated with the percentage of SWS or other CR proxies (all *p* values > 0.1). Between-sex differences were observed in sleep, cognition and CR proxies with women showing a greater proportion of SWS (*t* = 2.83, *p* = 0.006), higher episodic memory composite scores (*t* = 4.39, *p* < 0.001) and lower education (*t* = −2.9, *p* = 0.004), CAQ total (*t* = −2.13, *p* = 0.035), CAQ mid-life (*t* = −3.2, *p* = 0.003), CAQ late life (*t* = −2.4, *p* = 0.017), LEQ total (*t* = −2.18, *p* = 0.032) and LEQ mid-life (*t* = −2.1, *p* = 0.04). The matrix of simple linear regressions, assessing the associations between demographics, sleep, cognition and cognitive reserve proxies, is provided in Supplementary data (Supplementary Figure 1).

**Table 1:** Participants’ characteristics (*n* = 135).

	**Mean (SD)**	**Range**
*Demographics*		
Age, years	69.4 (3.8)	65–84
Women, *n* (%)	83 (61.5%)	NA
*Sleep (polysomnography)*		
Total sleep time (TST), min	360 (64.3)	192–550.5
Sleep latency, min	20.8 (13.8)	0–88.5
Wake after sleep onset, min	87.4 (49.1)	12–230
N1, % TST	13.8 (7.6)	3.5–43.9
N2, % TST	48.5 (8.7)	24.4–69.7
N3 (SWS), % TST	19.4 (9.5)	0–48.1
REM sleep, % TST	18.3 (5.6)	0–33.3
Sleep efficiency, %	77 (10.3)	49.6–96.6
Apnea-hypopnea index^*^	25.3 (14.8)	0.7–75.5
*Composite cognitive scores*		
Executive function	0 (1)	−3.04–2.47
Episodic memory	0 (1)	−2.81–1.93
*Cognitive reserve proxies*		
Education, years	13.2 (3.1)	7–22
Cognitive Activities Questionnaire (CAQ) - Total	3.2 (0.6)	1.5–4.5
Lifetime of Experiences Questionnaire (LEQ) - Total	97.9 (17.9)	56.4–143.1

Abbreviations: NA: not applicable; REM: rapid-eye movement; SD: standard deviation; SWS: slow wave sleep; TST: total sleep time. ^*^Missing data for 8 participants.

### Associations between SWS and cognition

**Figure 1:**
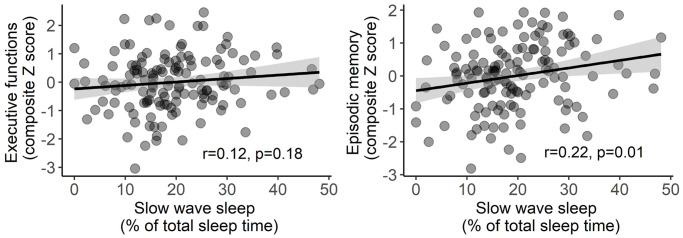
**Associations between slow wave sleep and both executive function (left) and episodic memory (right) composite scores.** Statistical values were obtained from simple linear regressions, without covariates. Shaded area represents 95% confidence intervals.

Multiple linear regressions revealed no association between the proportion of SWS and the executive function composite score (standardized β coefficient = 0.12, *p* = 0.18; Figure 1), while a significant positive association was found between the proportion of SWS and the episodic memory composite score (standardized β coefficient = 0.22, *p* = 0.01; Figure 1). Of note, while the association with episodic memory disappeared when controlling for age and sex (standardized β coefficient = 0.13, *p* = 0.11), exploratory interactions revealed that neither age nor sex modulated the relationship between SWS and composite cognitive scores (Age*SWS: *p* = 0.64 for executive function, and *p* = 0.44 for episodic memory; Sex*SWS: *p* = 0.07 and *p* = 0.36 for executive function and episodic memory composite scores, respectively).

### Interaction between CR proxies and SWS on cognition

**Figure 2:**
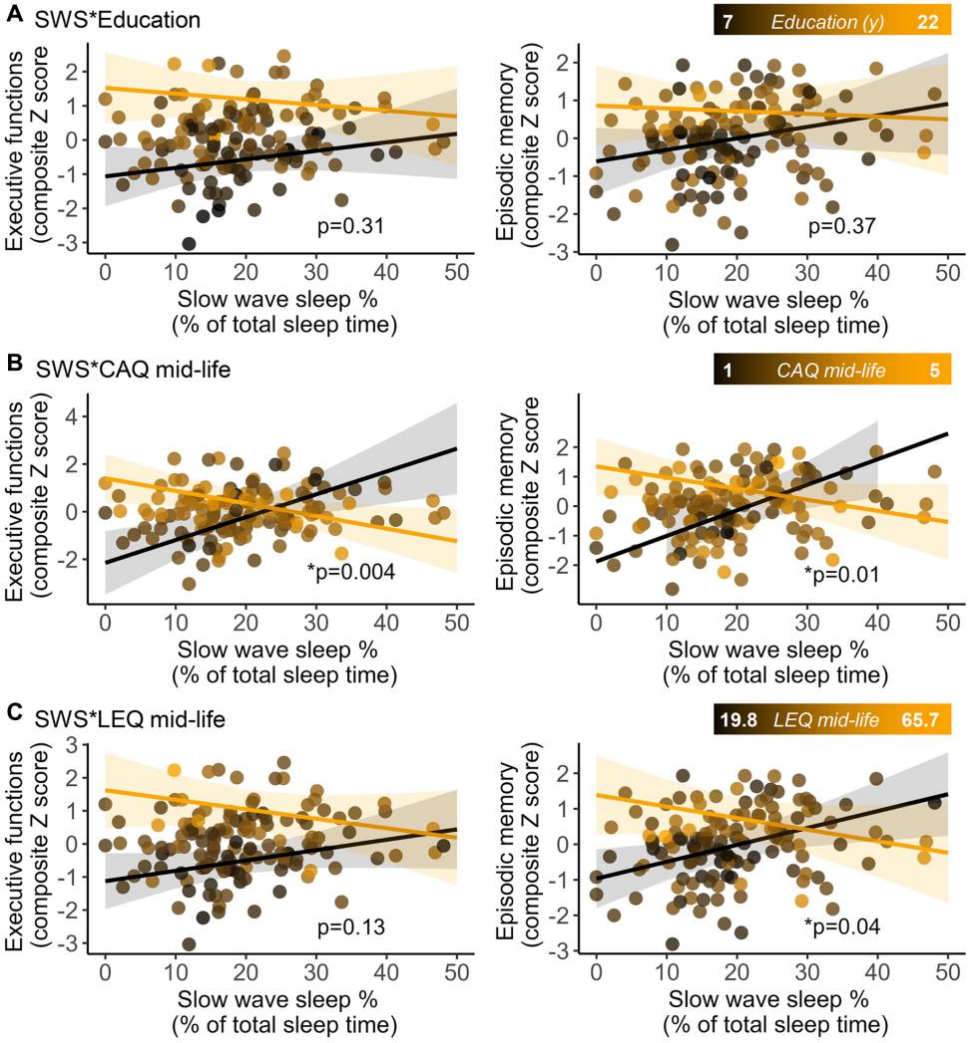
**Interactions between cognitive reserve proxies.** Education (**A**) Cognitive Activities Questionnaire for the mid-life period (**B**) and Lifetime of Experiences Questionnaire for the mid-life period (**C**) and slow wave sleep (SWS) on executive function (left panels) and episodic memory (right panels) composite scores. Marginal effect slopes were derived from the minimum (black) and the maximum (orange) value of the corresponding cognitive reserve proxy. Shaded areas represent 95% confidence intervals. Statistical values refer to the interactive term (SWS*CR proxy) and were obtained from general linear models, adjusted for age and sex.

To evaluate the moderating effect of CR on the link between SWS and cognition, separated multiple linear regression were used to assess the interaction between each CR proxies (total and period-specific scores) and SWS on each cognitive composite score, controlling for age and sex (Table 2 for full statistics). We did not find any interaction between education and SWS on executive function (*p* = 0.31) or episodic memory (*p* = 0.37; Figure 2ª). For executive function, we found an interaction between the CAQ total score and SWS (Standardized β coefficient = −0.38, *p* = 0.01), as well as between the CAQ mid-life score and SWS (Standardized β coefficient = −0.36, *p* = 0.004; Figure 2B). Similarly, we found a moderating effect of the CAQ total score (Standardized β coefficient = −0.37, *p* = 0.01), the CAQ early-life score (Standardized β coefficient = −0.3, *p* = 0.02), the CAQ mid-life score (Standardized β coefficient = −0.3, *p* = 0.01; Figure 2B) and the LEQ mid-life score (Standardized β coefficient = −0.02, *p* = 0.04; Figure 2C) on the association between SWS and episodic memory. These results survived false discovery rate (FDR) correction, except for the moderating effect of the LEQ mid-life score on the association between SWS and episodic memory (FDR adjusted *p* value = 0.11) (Table 2). For all interaction analyses, effects were such that individuals with higher CR (i.e., higher CAQ or LEQ scores) were able to maintain good executive and episodic memory performance when the proportion of SWS was low, while in individuals with lower CR, lower amounts of SWS were associated with poorer cognitive performance.

**Table 2:** Interactions between cognitive reserve proxies and slow wave sleep on cognition adjusted for age and sex.

**Interaction term**	**Executive function**					**Episodic memory**				
	**Standardized β coefficient (95% CI)**	***p* value (unadjusted)**	***P* value (FDR adjusted)**	**R^2^**	***F* value**	**Standardized β coefficient (95% CI)**	***p* value (unadjusted)**	***P* value (FDR adjusted)**	**R^2^**	***F* value**
SWS*Education	−0.08 (−0.24 0.08)	0.31	0.47	0.22	7.09	−0.07 (−0.24 0.09)	0.37	0.51	0.20	6.48
SWS*CAQ-Total	**−0.22 (−0.39 −0.06)**	**0.01**	**0.05**	**0.19**	**6.01**	**−0.22 (−0.38 −0.06)**	**0.01**	**0.05**	**0.24**	**7.97**
SWS*CAQ-Early	−0.17 (−0.35 0.01)	0.07	0.16	0.16	4.78	**−0.22 (−0.40 −0.04)**	**0.01**	**0.05**	**0.22**	**7.17**
SWS*CAQ-Mid-life	**−0.25 (−0.42 −0.08)**	**0.004**	**0.05**	**0.15**	**4.67**	**−0.21 (−0.37 −0.05)**	**0.01**	**0.05**	**0.23**	**7.65**
SWS*CAQ-Late	−0.15 (−0.32 0.02)	0.08	0.16	0.20	6.43	−0.15 (−0.32 0.01)	0.07	0.16	0.22	7.28
SWS*LEQ-Total	−0.03 (−0.20 0.15)	0.77	0.90	0.20	6.42	−0.10 (−0.28 0.07)	0.23	0.37	0.24	7.94
SWS*LEQ-Young	0.02 (−0.16 0.20)	0.83	0.90	0.17	5.13	−0.01 (−0.18 0.16)	0.9	0.90	0.22	7.08
SWS*LEQ-Mid-life	−0.11 (−0.24 0.03)	0.13	0.23	0.18	5.53	**−0.14 (−0.28 −0.01)**	**0.04**	**0.11**	**0.22**	**7.16**
SWS*LEQ-Late	−0.02 (−0.19 0.16)	0.85	0.90	0.14	4.13	−0.07 (−0.23 0.10)	0.44	0.56	0.24	7.99

Results in bold were considered significant at *p* < 0.05 (unadjusted). Abbreviations: LEQ: Lifetime of Experiences Questionnaire; CAQ: Cognitive Activities Questionnaire; SWS: slow wave sleep; CR: cognitive reserve; CI: confidence interval.

We performed additional analyses to ascertain the robustness of our findings while considering the influence of AHI, continuous positive airway pressure (CPAP) treatment at inclusion, adaptation night to the device, sex differences or potential outliers. Results remained unchanged, but did not survive FDR correction, when adjusting for AHI (Table 3). We replicated the analyses in individuals with no or mild apnea-hypopnea index (i.e., AHI < 15 events per hour of sleep; AHI-, *n* = 32) and individuals with moderate to severe sleep apnea (i.e., AHI ≥ 15 events per hour of sleep; AHI+, *n* = 95), the interaction between CAQ midlife and SWS on executive functions remained similar in the AHI- group while non-significant trends were found in the AHI+ group (Supplementary Table 1). The main results were unchanged when excluding participants with CPAP treatment at inclusion (*n* = 3) (Supplementary Table 2). When analyzing separately participants with an adaptation night (*n* = 88), interactions remained significant between CAQ (total and early-life scores) and SWS on episodic memory (Supplementary Table 3). In participants without an adaptation night (*n* = 47), results remained significant for the interactions between CAQ (total, early and mid-life scores) and SWS on executive function (Supplementary Table 3). Although analyses were adjusted for sex, we also performed additional interaction analyses in men and women separately and found that the interactions effects remained significant only in women (Supplementary Table 4). Finally, removing 5 outliers, deviating by +/− 3 standard deviations from the sample’s mean for at least one variable, have minimal effects on the main findings (Supplementary Table 5).

**Table 3:** Interactions between cognitive reserve proxies and slow wave sleep on cognition adjusted for age, sex and AHI.

**Interaction term**	**Executive function**					**Episodic memory**				
	**Standardized β coefficient (95% CI)**	***p* value (unadjusted)**	***P* value (FDR adjusted)**	**R^2^**	***F* value**	**Standardized β coefficient (95% CI)**	***p* value (unadjusted)**	***P* value (FDR adjusted)**	**R^2^**	***F* value**
SWS*Education	−0.07 (−0.23 0.09)	0.4	0.55	0.21	5.30	−0.09 (−0.26 0.08)	0.31	0.46	0.19	4.77
SWS*CAQ-Total	**−0.20 (−0.37 −0.03)**	**0.02**	**0.08**	**0.19**	**4.76**	**−0.21 (−0.38 −0.05)**	**0.01**	**0.08**	**0.23**	**5.90**
SWS*CAQ-Early	−0.16 (−0.35 0.03)	0.09	0.22	0.17	4.07	**−0.23 (−0.42 −0.04)**	**0.02**	**0.08**	**0.21**	**5.38**
SWS*CAQ-Mid-life	**−0.24 (−0.41 −0.07)**	**0.01**	**0.08**	**0.16**	**3.81**	**−0.21 (−0.38 −0.05)**	**0.01**	**0.08**	**0.22**	**5.71**
SWS*CAQ-Late	−0.13 (−0.30 0.04)	0.14	0.29	0.20	5.13	−0.15 (−0.32 0.03)	0.1	0.22	0.21	5.38
SWS*LEQ-Total	−0.01 (−0.19 0.17)	0.91	0.96	0.19	4.81	−0.11 (−0.29 0.07)	0.21	0.35	0.23	5.89
SWS*LEQ-Young	0.02 (−0.16 0.21)	0.79	0.95	0.17	4.07	−0.01 (−0.19 0.16)	0.87	0.96	0.21	5.27
SWS*LEQ-Mid-life	−0.10 (−0.24 0.05)	0.18	0.32	0.17	4.05	**−0.16 (−0.30 −0.01)**	**0.03**	**0.10**	**0.21**	**5.34**
SWS*LEQ-Late	−0.004 (−0.18 0.17)	0.96	0.96	0.14	3.39	−0.06 (−0.23 0.11)	0.46	0.59	0.23	5.94

Results in bold were considered significant at *p* < 0.05 (unadjusted). Abbreviations: LEQ: Lifetime of Experiences Questionnaire; CAQ: Cognitive Activities Questionnaire; SWS: slow wave sleep; CR: cognitive reserve; CI: confidence interval; AHI: apnea-hypopnea index.

## DISCUSSION AND CONCLUSION

In this study, we assessed the association between SWS and cognition in a cohort of cognitively unimpaired older adults and examined whether CR, assessed using different proxies reflecting mental activities over the life-course, could modulate these links. Our results show that the association between SWS and executive function and episodic memory performance differs according to CR, and this result seems more robust when considering the practice of cognitive/mental activities during mid-life.

Our analyses showed a positive, yet not robust, association between the proportion of SWS and episodic memory, while no association was found with executive performance. These findings support the major role of this sleep stage in memory processes [33]. Our results are also consistent with a series of studies showing a link between sleep and cognitive performance in older adults [7, 8, 12]. However, this association is not consistently found [7, 8, 19]. The effect of sleep on cognitive functioning appears to be less robust in older than in young adults [19], possibly because older adults may better tolerate the effects of sleep deprivation than young adults [20]. Indeed, older adults may have developed compensatory mechanisms that allow them to be less sensitive to the deleterious effect of sleep disturbances on cognition. In that context, CR appears as an interesting framework to explain the heterogeneous associations between SWS and cognition in older adults [21]. We found that individuals scoring higher on CR proxies were able to maintain good executive and episodic memory performance when facing sleep disturbances, expressed by a lower proportion of SWS. In contrast, older individuals with a low CR were more vulnerable to the effects of poor sleep (i.e., low amounts of SWS) and/or were unable to compensate for it, resulting in poorer cognitive performance. These results are consistent with those of studies showing that CR modulates the impact of poor sleep quality on cognition in older adults [29–31]. However, these studies used CR proxies that, while very commonly used, are relatively basic and represent an early construct of CR (education or IQ). In our study, we used more sophisticated and detailed questionnaires, allowing to characterize more precisely the influence of CR on the association between sleep and cognition. In particular, we assessed exposure to stimulating activities throughout the life-course to determine whether there are some critical periods to practice activities to promote successful aging. We did not replicated the previous results showing a protective effect of education, which may be partly explained by the fact that we had a slightly reduced range as, for instance, only individuals with at least 7 years of education were included in our study (compared with 3 to 22 years in [31]; and 3 to 27 years in [30]). Therefore, it is possible that the exclusion of individuals with lower levels of education in our study reduced the likelihood of finding an association. While CR is considered to be a dynamic construct that can evolve and grow over time, education is a static measure that is, therefore, not optimal to approximate CR. Indeed, mounting evidence points to the interest of considering multiple aspects of CR, especially its dynamics, in the context of aging and dementia [23, 28, 37, 38]. CR can still be nurtured during mid- and late-life, so that individuals, regardless of their level of education, can still build CR from other mentally stimulating activities and/or later in life. This view is supported by the fact that when using more refined proxies (i.e., questionnaires about cognitive activities at different life periods), we found that CR built-up during early life significantly modulated the association between SWS and cognition. However, this effect was observed only when using the CAQ as a proxy of CR, and not with the LEQ. This can be explained by the fact that the LEQ-Young adulthood score is strongly influenced by the level of education (i.e., the specific sub-score of the LEQ young adulthood is entirely based on education), while the CAQ focuses on cognitive engagement independently from education attainment (e.g., reading, writing letters, playing games). Our data also highlight the importance of mid-life cognitive and mentally stimulating activities to build-up CR, at least to help compensate for the effect of sleep disturbances in older-age on cognition. Interestingly, this was replicated for our two measures of cognition (i.e., executive function and episodic memory) with different CR proxies (CAQ and, only for episodic memory prior FDR-correction, LEQ) that assess complementary aspects of mental exposures such as cognitive activities and occupational complexity in mid-life. Therefore, exposure to several stimulating activities at mid-life may be one of the factors promoting successful aging and counteracting the effects of sleep changes in older-age on cognition. The importance of mid-life period on dementia risk has largely been shown when investigating the influence of cardiovascular risk factors [39–41]. Our study suggests that mid-life could also be a critical period to promote CR through greater engagement in cognitive activity. This aligns with previous evidence underlying the crucial role of midlife activities, including cognitive and socio-intellectual activities, to promote healthy cognitive aging [42–44]. These studies support the importance of lifelong engagement in stimulating activities. Surprisingly, however, CR built during late life does not appear to be sufficient to counteract the impact of poor sleep quality on cognitive performance in our study. It is possible that cognitive/mental stimulations should occur over a sufficient period of time to have a significant impact on cognition, and that our assessments of late-life CR (over one or a few years) have not been long enough to reveal such effects. Future studies in older participants might allow to further investigate this question.

Interestingly, we found that the moderating effects of CR were observed only in women. Considering that several studies have shown greater cognitive resilience against AD pathology or age-related changes in women than in men [45–47], it is possible that women also have higher cognitive resilience against age-related sleep changes than men. It is also possible that statistical power was not sufficient in men or that women, considering their differences from men in sleep, cognition and CR proxies, drive the findings. This should be further addressed in future studies with larger sample size.

Our study has several strengths. Analyses were conducted in a large sample of older participants who underwent a detailed cognitive assessment and polysomnography, the gold-standard for an objective sleep assessment. We also combined a classically used CR proxy (education) and more fine-grained proxies reflecting the exposure to stimulating activities over the life-course. The cognitive assessment included several tasks allowing us to investigate both executive functioning and episodic memory, particularly relevant as they are known to be sensitive to aging and dementia. Finally, most of the findings remained significant after applying FDR correction. Nonetheless, we could only partially replicate our findings when controlling for or splitting individuals according to their AHI. It could be due to a reduced sample size or collinearity in the model controlling for AHI (i.e., AHI and SWS are correlated, r = −0.3, *p* < 0.001); this could remove part of the sleep variability that we are interested in. A few limitations must be acknowledged. First, retrospective assessment of early/mid-life experiences is likely to be influenced by retrospective memory imprecisions, which could however be limited by the inclusion of cognitively unimpaired adults only in this study; alternatively individuals with overall better memory at the time of assessment may also recall life events more precisely, thus potentially obtaining CR scores that are more reliable. Also, we analyzed the different life periods independently, in separated models. As lifestyle (e.g., cognitive activities) at a given time is likely to be associated with activities conducted during the other life periods, the specificity of the results to a single period should be interpreted with caution. For instance, whether the effect of CAQ-total is mainly driven by mid-life cognitive engagement or results from the combination of lifelong engagement remains to be fully understood. Second, 34.8% of the participants did not have an adaptation night to the polysomnography. While these participants did not show a major decrease in sleep parameters compared to those who had an adaptation night (*p* = 0.09 and *p* = 0.83 for SWS% and AHI, respectively), we cannot exclude that this might have influenced sleep quality of the night recorded. Finally, future studies including neuroimaging measures would allow to capture the brain mechanisms of CR that could protect against the effects of sleep changes in older-age on cognition.

We showed, for the first time in a large sample of older adults, that individuals with a higher CR were able to maintain good executive and episodic memory performance even in case of low amounts of SWS measured objectively by polysomnography. In contrast, older adults with a low CR were more vulnerable to SWS reduction and showed poor cognitive performance. These results suggest that CR may protect against the negative effect of sleep changes in older-age on cognitive performance, and that mid-life could be a potential period of interest which remains to be further investigated in prospective studies. These findings are important to understand the factors promoting successful aging and suggest that the deleterious impact of sleep disturbances could be counteracted by an enriched lifestyle. This will help to design non-pharmacological interventions to promote successful aging and counter age-related sleep changes.

## MATERIALS AND METHODS

### Participants

We included 135 cognitively unimpaired older adults from the baseline visit of the Age-Well randomized controlled trial (see [34] for the detailed protocol), sponsored by the French National Institute of Health and Medical Research (INSERM). Participants were native French speakers, recruited from the general population, aged 65 years old and over, and retired for at least one year. They were educated for at least 7 years and performed within the normal range for their age, sex and educational level on standardized cognitive tests from the diagnosis battery [34], including the MMSE [48], RL-RI16 [49] and the Modified Card Sorting Task [50] (z score > −1.65 according to age, sex and education). They had no evidence of major neurological or psychiatric disorders, no history of cerebrovascular disease, chronic disease or acute unstable illness and no current medication that might interfere with cognitive functioning [34]. At baseline, over a maximum period of 3 months, participants underwent a comprehensive neuropsychological and lifestyle assessment, as well as an ambulatory polysomnography (PSG). Sleep quality was not part of the inclusion and exclusion criteria. Thus, participants with various levels of sleep quality were included, allowing to have variability in sleep parameters. All participants gave their written informed consent to participate in the study, which was approved by local ethics committee (CPP Nord-Ouest III, Caen; trial registration number: EudraCT: 2016-002441-36; IDRCB: 2016-A01767-44; ClinicalTrials.gov Identifier: NCT02977819). Participants’ characteristics are presented in Table 1.

### Sleep

Participants underwent a polysomnography (PSG) at home using an ambulatory device (Siesta^®^, Compumedics, Australia), as previously described [51]. Briefly, the PSG examination included the recording of the electroencephalogram (EEG), electrooculogram (EOG), chin electromyogram (EMG), electrocardiogram (ECG), respiratory movements using thoracic and abdominal belts, respiratory airflow using nasal and oral thermistors, and oxygen saturation using a finger pulse oximeter. For the EEG recording, twenty electrodes were placed over the scalp according to the international 10–20 system (Fp1, Fp2, F3, F4, F7, F8, Fz, C3, C4, Cz, T3, T4, P3, P4, Pz, O1, O2, vertex ground, and a bi-mastoid reference), with impedances kept below 5 kΩ. The EEG signal was digitalized at a sampling rate of 256 Hz, high-pass and low-pass filters were applied, respectively at 0.3 Hz and 35 Hz. The recordings were then visually scored by two experts (C.A., S.R.) in 30s epochs according to the American Academy of Sleep Medicine scoring rules [52]. Standard sleep parameters and the apnea-hypopnea index (AHI) were computed. Over the 135 participants, 65.2% (88 participants) benefited from two PSG recordings and the first one (adaptation night) was not analyzed, while the remaining 34.8% (47 participants) only had one night of PSG, which was included in the analyses. In addition, for eight participants, respiratory/oximetry data were not fully exploitable. As a consequence, the AHI is missing for those participants. Considering its particular sensitivity to aging, and to limit multiple comparisons, we focused our analyses on SWS (proportion of SWS relative to total sleep time). In this study, 3 participants had a continuous positive airway pressure (CPAP) treatment for sleep apnea at inclusion. Sleep data are summarized in Table 1.

### Composite cognitive scores

Participants underwent a detailed neuropsychological examination [34] which was independent from the diagnostic neuropsychological battery used for participants’ inclusion (as detailed above). From this examination, executive function and episodic memory composite scores were computed and were selected in the present study as they are both particularly sensitive to aging and AD [35, 53] and closely related to sleep quality [54, 55].

The executive function composite score was created from four established measures of executive function: the Digit Span backward (raw score), Trail Making Test part B (response time), Stroop interference index (response time for interference-naming items) and Letter Fluency (number of words beginning with P letter provided in 2 minutes). For each test, z-scores were computed using the total sample’s mean score and standard deviation. Trail Making Test and Stroop scores were reversed so that higher scores always indicate better performance. The executive function composite score corresponds to the unweighted average of these four z-scores.

The episodic memory composite score was created from two established measures of episodic memory: The California Verbal Learning Test (CVLT) and the Wechsler Memory Scale IV Logical Memory, Story B (WMS IV) More specifically, the CVLT scores included the sum of trials 1–5, immediate free recall and delayed free recall, while the Logical Memory scores included immediate and delayed free recall. As for the executive composite, each sub-score was standardized using Age-Well baseline sample average score and standard deviation. The episodic memory composite score corresponds to the unweighted average of these five z-scores.

Finally, to facilitate data interpretation, the cognitive composite scores were re-standardized (subtracting the average of the total sample and dividing by standard deviation of the total sample). In both cases, higher scores indicated better performance.

### Cognitive reserve proxies

Participants completed the French versions of the Cognitive Activities Questionnaire (CAQ) [27] and the Lifetime of Experiences Questionnaire (LEQ) [28, 36], which were used as CR proxies. In order to compare the results with existing studies, education (in years) was also used as a CR proxy.

The CAQ assesses cognitive activities for different life periods: at age 6, age 12, age 18, age 40, and for the current period [27]. Responses were provided on a 5-point scale corresponding to the frequency of participation, ranging from 1 (once a year or less) to 5 (every day or almost every day). For each participant, we calculated three sub-scores: early life (average of the 6y, 12y and 18y periods), mid-life (average of the 40y period), late life (average of the current period), and a lifetime cognitive activity score (average of all age periods). These different scores are hereafter referred to as CAQ early life, CAQ mid-life, CAQ late life and CAQ total, respectively.

The LEQ assesses complex mental activities (e.g., education, occupational complexity, cognitive, social and physical activities) across 3 life periods: young adulthood (13–30 years), mid-life (30–65 years) and late life (from 65 years to present date) [28]. Each life period comprises a specific (i.e., activities specific to this period, including for instance education in early life and occupation at mid-life) and a non-specific (i.e., activities that can be conducted at any life period, including for instance visits to family, physical activities, reading or traveling) score. Specific and non-specific scores were summed to give a sub-score for each period (LEQ young adulthood, LEQ mid-life and LEQ late life). Then, these three sub-scores were summed to obtain a LEQ total score.

For both questionnaires, higher scores reflect greater engagement in cognitive and/or complex mental activities, therefore greater CR.

### Statistical analyses

First, we assessed the associations between age, sex and our measures of interest (% SWS, composite cognitive scores and CR proxies) using either correlations for continuous variables (age) or *t*-tests for discrete variables (sex). Second, we performed multiple linear regressions using each composite cognitive score as a dependent variable and the percentage of SWS as independent variable. The same analyses were repeated including age and sex as covariates.

Third, using multiple linear regressions, we tested the interaction between each CR proxy (education, CAQ total, early life, mid-life, late life and LEQ total, young, mid-life and late life; entered separately in independent models) and SWS on each cognitive composite score, adjusting for age and sex, as follows: Cognition ~ SWS * CR proxy + Age + Sex. Interaction models were replicated to address potential confounds and assess the robustness of our findings while considering the influence of AHI, continuous positive airway pressure (CPAP) treatment at inclusion, adaptation night to the PSG device, sex differences or potential outliers. In order to consider sleep apnea, which may have an impact on both sleep architecture and cognition, we repeated our interaction analyses adjusting for AHI and by splitting the sample with a AHI clinical cutoff value of ≥ 15 [56], allowing to distinguish individuals with no or mild apnea (AHI < 15, referred hereafter as AHI- group, *n* = 32) from those with moderate to severe sleep apnea (AHI ≥ 15, AHI+ group, *n* = 95). Models were also conducted after the exclusion of participants with a CPAP treatment and in participants who underwent an adaptation night and those who did not underwent and adaptation night separately. To explore the potential influence of sex differences on our results, we repeated these interaction analyses separately in men (*n* = 52) and women (*n* = 83). Finally, analyses were replicated after the exclusion of outliers (i.e., participants with measurements +/− 3 standard deviation from the sample’s mean on at least one variable).

Analyses were performed using the R 4.0.2 software, and were considered significant at *p* ≤ 0.05 unadjusted. All models satisfied statistical assumptions for linear regression model.

### Data availability

Data is available on request following a formal data sharing agreement and approval by the consortium and executive committee. The data sharing request form can be downloaded at https://silversantestudy.eu/2020/09/25/data-sharing/.

### Medit-Ageing Research Group

Florence Allais, Eider M Arenaza Urquijo, Julien Asselineau, Pierre Champetier, Anne Chocat, Sophie Dautricourt, Robin de Flores, Eglantine Ferrand Devouge, Eric Frison, Valérie Lefranc, Antoine Lutz, Géraldine Poisnel, Anne Quillard, Eric Salmon, Edelweiss Touron.

## Supplementary Materials

Supplementary Figure 1

Supplementary Tables
