# Supplementary material for: Effect of cognitive reserve on the association between slow wave sleep and cognition in community-dwelling older adults
Source: Aging (Albany NY). 2023 Sep 28;15(18):9275–92. doi: 10.18632/aging.204943 (PMC10564409; doi:10.18632/aging.204943)
Supplement: Supplementary Figure 1 [file aging-15-204943-s001.pdf]

SUPPLEMENTARY FIGURE

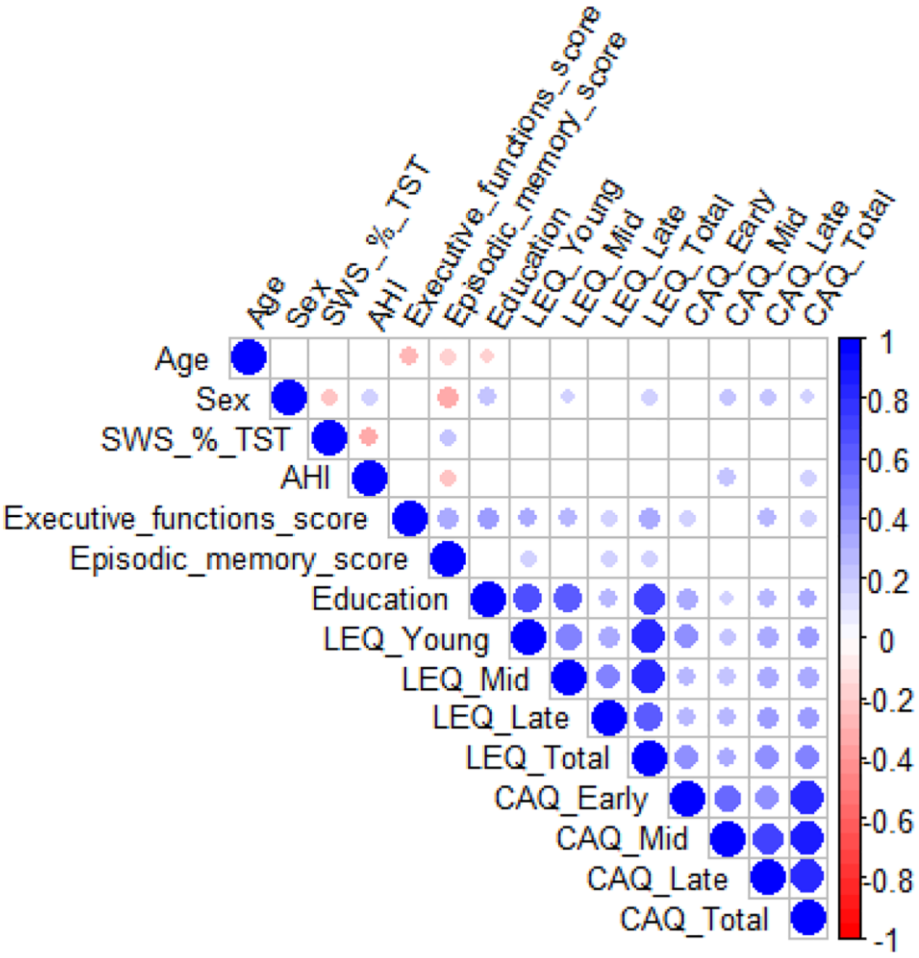

Supplementary Figure 1. Simple linear regressions matrix between demographics, sleep, cognition and cognitive reserve proxies. Standardized β coefficients are indicated in blue (positive) or red (negative) or white when $p > 0.05$. Numerical values of Sex are F = 0 and M = 1. Abbreviations: LEQ: Lifetime of Experiences Questionnaire; CAQ: Cognitive Activities Questionnaire; SWS: Slow Wave Sleep; TST: total sleep time; AHI: Apnea Hypopnea Index. Mid refers to midlife.
