# Supplementary material for: Effect of cognitive reserve on the association between slow wave sleep and cognition in community-dwelling older adults
Source: Aging (Albany NY). 2023 Sep 28;15(18):9275–92. doi: 10.18632/aging.204943 (PMC10564409; doi:10.18632/aging.204943)
Supplement: Supplementary Tables [file aging-15-204943-s002.pdf]

SUPPLEMENTARY TABLES

Supplementary Table 1. Interaction between cognitive reserve proxies and slow wave sleep on cognition, adjusted for age and sex, in AHI+ (*n* = 95) and AHI- (*n* = 32).

Interaction term	AHI+ (*n* = 95)							
	Executive function				Episodic memory			
	Standardized β coefficient (95% CI)	*p* value	R^2	F-test	Standardized β coefficient (95% CI)	*p* value	R^2	F-test
SWS*Education	−0.09 (−0.27 0.1)	0.35	0.23	5.41	−0.08 (−0.26 0.1)	0.40	0.22	4.92
SWS*CAQ-Total	−0.17 (−0.36 0.02)	0.09	0.18	4.03	−0.16 (−0.35 0.02)	0.08	0.24	5.50
SWS*CAQ-Early	−0.13 (−0.34 0.08)	0.23	0.17	3.65	−0.16 (−0.36 0.05)	0.13	0.22	5.10
SWS*CAQ-Mid-life	−0.18 (−0.38 0.02)	0.08	0.15	3.21	−0.15 (−0.34 0.04)	0.11	0.23	5.20
SWS*CAQ-Late	−0.16 (−0.37 0.04)	0.12	0.20	4.47	−0.15 (−0.35 0.05)	0.13	0.23	5.44
SWS*LEQ-Total	−0.04 (−0.25 0.18)	0.72	0.16	3.33	−0.1 (−0.31 0.1)	0.31	0.23	5.27
SWS*LEQ-Young	0.02 (−0.2 0.24)	0.87	0.16	3.28	−0.03 (−0.24 0.18)	0.75	0.21	4.72
SWS*LEQ-Mid-life	−0.1 (−0.27 0.08)	0.27	0.15	3.09	−0.12 (−0.28 0.04)	0.14	0.23	5.22
SWS*LEQ-Late	0 (−0.2 0.2)	1.00	0.13	2.72	−0.07 (−0.25 0.12)	0.50	0.23	5.25
**Interaction term**	**AHI- (*n* = 32)**							
	**Executive function**				**Episodic memory**			
	**Standardized β coefficient (95% CI)**	***p* value**	R^2	**F-test**	**Standardized β coefficient (95% CI)**	***p* value**	R^2	**F-test**
SWS*Education	−0.01 (−0.45 0.43)	0.97	0.21	1.40	−0.15 (−0.61 0.31)	0.52	0.22	1.50
SWS*CAQ-Total	−0.4 (−0.87 0.06)	0.09	0.27	1.89	−0.24 (−0.74 0.26)	0.33	0.26	1.79
SWS*CAQ-Early	−0.45 (−0.97 0.07)	0.09	0.25	1.77	−0.37 (−0.92 0.18)	0.18	0.26	1.78
SWS*CAQ-Mid-life	**−0.48 (−0.9 −0.06)**	**0.03**	**0.24**	**1.67**	−0.28 (−0.71 0.16)	0.20	0.26	1.84
SWS*CAQ-Late	−0.05 (−0.43 0.34)	0.81	0.25	1.69	0.03 (−0.38 0.44)	0.88	0.27	1.88
SWS*LEQ-Total	0.06 (−0.26 0.37)	0.72	0.55	6.25	−0.1 (−0.48 0.29)	0.61	0.37	3.06
SWS*LEQ-Young	−0.19 (−0.59 0.21)	0.35	0.36	2.91	−0.21 (−0.62 0.2)	0.31	0.39	3.39
SWS*LEQ-Mid-life	0.01 (−0.31 0.32)	0.96	0.41	3.67	−0.29 (−0.65 0.08)	0.12	0.29	2.08
SWS*LEQ-Late	−0.03 (−0.43 0.36)	0.86	0.34	2.68	0.11 (−0.28 0.5)	0.56	0.42	3.84

Results in bold were considered significant at $p \leq 0.05$. Abbreviations: LEQ: Lifetime of Experiences Questionnaire; CAQ: Cognitive Activities Questionnaire; SWS: slow wave sleep; CR: cognitive reserve; CI: confidence interval; AHI: Apnea Hypopnea Index. Dichotomization between AHI+ and AHI- was based on the AHI clinical cutoff value of ≥15.

Supplementary Table 2. Interaction between cognitive reserve proxies and slow wave sleep on cognition, adjusted for age and sex, excluding participants with CPAP treatment (*n* = 3).

Interaction term	Executive function				Episodic memory			
	Standardized β coefficient (95% CI)	*p* value	R^2	F-test	Standardized β coefficient (95% CI)	*p* value	R^2	F-test
SWS*Education	−0.06 (−0.22 0.1)	0.42	0.21	6.66	−0.07 (−0.24 0.09)	0.38	0.20	6.27
SWS*CAQ-Total	**−0.21 (−0.37 −0.04)**	**0.01**	**0.19**	**6.00**	**−0.22 (−0.38 −0.05)**	**0.01**	**0.24**	**7.76**
SWS*CAQ-Early	−0.16 (−0.34 0.02)	0.08	0.17	5.04	**−0.23 (−0.41 −0.04)**	**0.02**	**0.22**	**7.07**
SWS*CAQ-Mid-life	**−0.24 (−0.4 −0.07)**	**0.005**	**0.16**	**4.80**	**−0.21 (−0.37 −0.04)**	**0.01**	**0.23**	**7.44**
SWS*CAQ-Late	−0.13 (−0.3 0.03)	0.12	0.20	6.37	−0.15 (−0.32 0.02)	0.08	0.22	7.06
SWS*LEQ-Total	−0.01 (−0.19 0.16)	0.90	0.20	6.12	−0.11 (−0.29 0.06)	0.21	0.24	7.75
SWS*LEQ-Young	0.02 (−0.15 0.2)	0.79	0.17	5.19	−0.01 (−0.19 0.16)	0.88	0.22	6.91
SWS*LEQ-Mid-life	−0.1 (−0.24 0.04)	0.17	0.17	5.16	**−0.16 (−0.3 −0.01)**	**0.03**	**0.22**	**7.07**
SWS*LEQ-Late	0 (−0.18 0.17)	0.96	0.14	4.27	−0.07 (−0.23 0.1)	0.44	0.24	7.79

Results in bold were considered significant at $p \leq 0.05$. Abbreviations: LEQ: Lifetime of Experiences Questionnaire; CAQ: Cognitive Activities Questionnaire; SWS: slow wave sleep; CR: cognitive reserve; CI: confidence interval; CPAP: continuous positive airway pressure. The analysis was conducted in 132 participants.

Supplementary Table 3. Interaction between cognitive reserve proxies and slow wave sleep on cognition, adjusted for age and sex, in participants with (*n* = 88) and without (*n* = 47) an adaptation night.

Interaction term	Participants with an adaptation night (*n* = 88)							
	Executive function				Episodic memory			
	Standardized β coefficient (95% CI)	*p* value	R^2	F-test	Standardized β coefficient (95% CI)	*p* value	R^2	F-test
SWS*Education	−0.08 (−0.27 0.12)	0.43	0.26	5.81	−0.07 (−0.26 0.13)	0.50	0.22	4.57
SWS*CAQ-Total	−0.15 (−0.36 0.05)	0.14	0.19	3.86	**−0.22 (−0.41 −0.03)**	**0.02**	**0.26**	**5.88**
SWS*CAQ-Early	−0.08 (−0.32 0.16)	0.53	0.17	3.30	**−0.25 (−0.47 −0.03)**	**0.03**	**0.26**	**5.70**
SWS*CAQ-Mid-life	−0.16 (−0.37 0.05)	0.13	0.15	2.96	−0.19 (−0.38 0)	0.06	0.25	5.43
SWS*CAQ-Late	−0.12 (−0.33 0.08)	0.24	0.24	5.22	−0.18 (−0.38 0.02)	0.07	0.25	5.50
SWS*LEQ-Total	−0.01 (−0.22 0.2)	0.91	0.25	5.39	−0.14 (−0.34 0.06)	0.17	0.26	5.71
SWS*LEQ-Young	0.02 (−0.18 0.22)	0.84	0.21	4.49	−0.07 (−0.26 0.13)	0.51	0.23	4.83
SWS*LEQ-Mid-life	−0.1 (−0.26 0.07)	0.24	0.22	4.52	−0.13 (−0.29 0.03)	0.10	0.25	5.37
SWS*LEQ-Late	0 (−0.23 0.23)	0.98	0.17	3.39	−0.11 (−0.32 0.1)	0.31	0.27	6.17
**Interaction term**	**Participants without adaptation night (*n* = 47)**							
	**Executive function**				**Episodic memory**			
	**Standardized β coefficient (95% CI)**	***p* value**	R^2	**F-test**	**Standardized β coefficient (95% CI)**	***p* value**	R^2	**F-test**
SWS*Education	−0.21 (−0.58 0.16)	0.26	0.17	1.74	0.01 (−0.4 0.42)	0.94	0.23	2.45
SWS*CAQ-Total	**−0.47 (−0.81 −0.12)**	**0.01**	**0.28**	**3.23**	−0.2 (−0.61 0.21)	0.33	0.24	2.57
SWS*CAQ-Early	**−0.34 (−0.66 −0.03)**	**0.03**	**0.23**	**2.47**	−0.17 (−0.54 0.2)	0.35	0.19	1.98
SWS*CAQ-Mid-life	**−0.54 (−0.87 −0.21)**	**0.002**	**0.30**	**3.59**	−0.3 (−0.69 0.09)	0.12	0.25	2.70
SWS*CAQ-Late	−0.14 (−0.45 0.17)	0.35	0.16	1.58	−0.04 (−0.38 0.29)	0.80	0.24	2.62
SWS*LEQ-Total	−0.07 (−0.47 0.34)	0.75	0.15	1.46	0.07 (−0.36 0.49)	0.76	0.25	2.81
SWS*LEQ-Young	0.06 (−0.36 0.49)	0.76	0.12	1.11	0.28 (−0.15 0.71)	0.20	0.31	3.70
SWS*LEQ-Mid-life	−0.19 (−0.49 0.1)	0.20	0.18	1.74	−0.17 (−0.5 0.16)	0.30	0.21	2.14
SWS*LEQ-Late	0.13 (−0.21 0.46)	0.44	0.16	1.58	0.06 (−0.3 0.43)	0.73	0.22	2.33

Results in bold were considered significant at $p \leq 0.05$. Abbreviations: LEQ: Lifetime of Experiences Questionnaire; CAQ: Cognitive Activities Questionnaire; SWS: slow wave sleep; CR: cognitive reserve; CI: confidence interval; CPAP: continuous positive airway pressure.

Supplementary Table 4. Interaction between cognitive reserve proxies and slow wave sleep on cognition, adjusted for age, in women (*n* = 83) and men (*n* = 52).

Interaction term	Women (*n* = 83)							
	Executive function				Episodic memory			
	Standardized β coefficient (95% CI)	*p* value	R^2	F-test	Standardized β coefficient (95% CI)	*p* value	R^2	F-test
SWS*Education	−0.17 (−0.42 0.07)	0.15	0.23	5.84	−0.19 (−0.44 0.05)	0.12	0.14	3.12
SWS*CAQ-Total	**−0.29 (−0.53 −0.05)**	**0.02**	**0.25**	**6.41**	**−0.28 (−0.52 −0.04)**	**0.02**	**0.19**	**4.48**
SWS*CAQ-Early	−0.19 (−0.43 0.05)	0.11	0.22	5.47	**−0.29 (−0.53 −0.05)**	**0.02**	**0.16**	**3.85**
SWS*CAQ-Mid-life	**−0.36 (−0.61 −0.11)**	**0.005**	**0.21**	**5.17**	**−0.3 (−0.54 −0.06)**	**0.02**	**0.18**	**4.17**
SWS*CAQ-Late	−0.21 (−0.46 0.04)	0.10	0.26	6.88	−0.15 (−0.41 0.11)	0.25	0.16	3.65
SWS*LEQ-Total	−0.12 (−0.41 0.18)	0.43	0.22	5.61	−0.21 (−0.5 0.08)	0.15	0.19	4.70
SWS*LEQ-Young	−0.07 (−0.32 0.18)	0.59	0.21	5.14	−0.13 (−0.38 0.12)	0.31	0.14	3.17
SWS*LEQ-Mid-life	−0.18 (−0.38 0.02)	0.07	0.24	6.04	**−0.2 (−0.4 −0.01)**	**0.04**	**0.18**	**4.35**
SWS*LEQ-Late	−0.14 (−0.42 0.15)	0.35	0.14	3.18	−0.16 (−0.43 0.1)	0.22	0.20	4.88
**Interaction term**	**Men (*n* = 52)**							
	**Executive function**				**Episodic memory**			
	**Standardized β coefficient (95% CI)**	***p* value**	R^2	**F-test**	**Standardized β coefficient (95% CI)**	***p* value**	R^2	**F-test**
SWS*Education	0.09 (−0.15 0.34)	0.44	0.24	3.72	0.07 (−0.18 0.32)	0.57	0.06	0.77
SWS*CAQ-Total	−0.12 (−0.39 0.15)	0.37	0.11	1.43	−0.2 (−0.45 0.06)	0.12	0.09	1.10
SWS*CAQ-Early	−0.18 (−0.49 0.13)	0.25	0.10	1.28	−0.17 (−0.47 0.12)	0.24	0.07	0.83
SWS*CAQ-Mid-life	−0.08 (−0.36 0.2)	0.56	0.08	1.04	−0.16 (−0.42 0.09)	0.21	0.07	0.89
SWS*CAQ-Late	−0.05 (−0.33 0.24)	0.74	0.13	1.72	−0.24 (−0.51 0.03)	0.08	0.10	1.32
SWS*LEQ-Total	0.1 (−0.13 0.32)	0.40	0.19	2.83	−0.03 (−0.25 0.2)	0.83	0.06	0.71
SWS*LEQ-Young	0.13 (−0.13 0.39)	0.32	0.13	1.79	0.12 (−0.12 0.37)	0.31	0.10	1.33
SWS*LEQ-Mid-life	0.01 (−0.21 0.23)	0.92	0.12	1.64	−0.12 (−0.33 0.09)	0.24	0.07	0.84
SWS*LEQ-Late	0.21 (−0.01 0.42)	0.07	0.30	4.95	0 (−0.24 0.23)	0.98	0.05	0.67

Results in bold were considered significant at $p \leq 0.05$. Abbreviations: LEQ: Lifetime of Experiences Questionnaire; CAQ: Cognitive Activities Questionnaire; SWS: slow wave sleep; CR: cognitive reserve; CI: confidence interval.

Supplementary Table 5. Interactions between cognitive reserve proxies and slow wave sleep on cognition adjusted for age and sex, without outliers (*n* = 5) derived from 3 standard deviations.

Interaction term	Executive function				Episodic memory			
	Standardized β coefficient (95% CI)	*p* value	R^2	F value	Standardized β coefficient (95% CI)	*p* value	R^2	F value
SWS*Education	−0.03 (−0.19 0.13)	0.74	0.18	5.57	−0.07 (−0.24 0.1)	0.40	0.23	7.25
SWS*CAQ-Total	**−0.19 (−0.36 −0.02)**	**0.03**	**0.17**	**5.10**	**−0.18 (−0.35 −0.01)**	**0.04**	**0.25**	**8.09**
SWS*CAQ-Early	−0.14 (−0.33 0.05)	0.15	0.14	4.02	−0.18 (−0.36 0.01)	0.07	0.23	7.39
SWS*CAQ-Mid-life	**−0.23 (−0.4 −0.06)**	**0.01**	**0.15**	**4.21**	**−0.18 (−0.34 −0.01)**	**0.04**	**0.24**	**7.85**
SWS*CAQ-Late	−0.11 (−0.28 0.05)	0.18	0.18	5.35	−0.13 (−0.29 0.04)	0.14	0.24	8.01
SWS*LEQ-Total	0.02 (−0.16 0.2)	0.80	0.19	5.68	−0.07 (−0.25 0.11)	0.43	0.25	8.13
SWS*LEQ-Young	0.08 (−0.1 0.26)	0.36	0.18	5.33	0 (−0.19 0.18)	0.98	0.23	7.56
SWS*LEQ-Mid-life	−0.09 (−0.26 0.08)	0.30	0.15	4.23	−0.15 (−0.32 0.02)	0.08	0.24	7.72
SWS*LEQ-Late	0.02 (−0.16 0.2)	0.81	0.13	3.77	−0.01 (−0.19 0.17)	0.92	0.25	8.32

Results in bold were considered significant at $p \leq 0.05$. Abbreviations: LEQ: Lifetime of Experiences Questionnaire; CAQ: Cognitive Activities Questionnaire; SWS: slow wave sleep; CR: cognitive reserve; CI: confidence interval. Results in orange are not significant anymore compare to the main table.
